# Displaced distal radius fractures in children, cast alone vs additional K-wire fixation: a meta-analysis

**DOI:** 10.1007/s00068-018-1011-y

**Published:** 2018-10-01

**Authors:** Alysia Sengab, Pieta Krijnen, Inger Birgitta Schipper

**Affiliations:** Department of Trauma Surgery, Leiden University Medical Center, Post zone K6-R, P.O. Box 9600, 2300 RC Leiden, The Netherlands

**Keywords:** Distal radius, Paediatric, Cast immobilization, K-wire fixation, Reduction, Redisplacement, Outcome, Range of motion, Complications

## Abstract

**Purpose:**

Displaced distal radius fractures in children are common and often treated by reduction and cast immobilization. Redisplacement occurs frequently and may be prevented by additional treatment with K-wire fixation after initial reduction. This meta-analysis aims to summarize available literature on this topic and determine if primary K-wire fixation is the preferred treatment for displaced distal radius fractures in children.

**Methods:**

A search in eight databases identified studies that compared cast immobilization alone to additional K-wire fixation as treatment for displaced paediatric distal radius fractures. The primary outcome was the redisplacement rate. Secondary outcomes were secondary reduction rate, range of motion and complications. This meta-analysis was performed according to the preferred reporting items for systematic reviews and meta-analysis (PRISMA) statement.

**Results:**

Three RCTs and 3 cohort studies, analysing 197 patients treated with cast immobilization alone and 185 with additional K-wire fixation, were included in this meta-analysis. Redisplacement occurred less frequently after additional K-wire fixation than after cast alone (3.8 versus 45.7%; OR 0.07, 95% CI 0.03–0.15). Secondary reduction was performed in 59.8% of the redisplaced fractures. Complications, other than redisplacement, occurred more often after additional K-wire fixation than after cast alone (15.7 versus 3.6%). Range of motion did not differ after both treatments.

**Conclusions:**

Additional K-wire fixation is a suitable treatment to prevent redisplacement and secondary operations after initial reduction of displaced distal radius fractures in children, but is associated with post-procedural complications. Additional K-wire fixation does not result in a better range of motion than cast immobilization alone. More research is needed to identify those patients who will benefit the most from K-wire fixation as a treatment for displaced distal radius fractures in children.

**Electronic supplementary material:**

The online version of this article (10.1007/s00068-018-1011-y) contains supplementary material, which is available to authorized users.

## Introduction

Distal radius fractures (DRFs) are amongst the most common fractures in children. They account for 19.9–35.8% of all paediatric fractures and are often treated with reduction and cast immobilization (RCI) [1–4]. Recent studies have shown, however, that redisplacement after RCI within the first 2 weeks after initial reduction occurs in 21–34% of cases [5–8]. To prevent redisplacement after initial reduction and to avoid the need for secondary treatment, displaced DRFs (DDRFs) can be treated with reduction and percutaneous K-wire fixation (KWF) before cast immobilization. Although additional KWF has shown to decrease redisplacement rates, it can also lead to complications such as pin-tract infection and neuropraxia [9–13]. The primary aim of this study was to summarise the available literature on this topic in a meta-analysis and compare the effects of RCI alone and additional KWF on the redisplacement rate of initially DDRFs. Other outcomes were the secondary reduction rate, complications and range of motion.

## Materials and methods

This meta-analysis was performed according to the ‘Preferred Reporting Items for Systematic reviews and Meta-Analyses: the PRISMA statement’ [14].

### Study selection

A literature search was performed in PubMed, Embase, Web of Science, Cochrane, CENTRAL, CINAHL, Academic Search Premier and Science Direct on 22nd of November 2016 and updated on 14th of November 2017. The search strategy was composed by an experienced medical librarian and included different synonyms of the keywords ‘Radius Fractures, Displaced, Child, Internal Fracture Fixation, Surgical Casts and Immobilization’ (see Appendix 1 in Supplementary Material for the exact search strategy).

Articles were selected independently by two reviewers (AS/PK) on (1) inclusion of skeletally immature patients (2) having a DRF (with or without distal ulnar fracture) with at least 50% bone width displacement or an angulation requiring manipulation, (3) treated with reduction and either above or below elbow cast immobilization alone (AEC, BEC) or additional KWF, and (4) compared outcomes for the two treatment options. (5) Treatment groups had to be comparable within studies, regarding patient and fracture related characteristics. (6) Articles had to be written in English. Articles were excluded if they also analysed other forearm fractures and results concerning DDRFs could not be extracted.

### Data extraction

From the included articles, two reviewers (AS/PK) independently extracted data on study characteristics (author, title, publication year, type of study, number of included patients), patient characteristics (age, gender), duration of follow-up, and outcomes (redisplacement in all patients and in patient subgroups, secondary reduction, ROM in degrees and complications). Authors of the included articles were asked for more information if presented data was insufficient.

### Statistical analysis

A meta-analysis using Review Manager 5.3 was performed, if the selected studies included comparable study groups and had applied similar data definitions. Treatment effects were estimated by computing the odds ratio (OR) with 95% confidence interval (CI) for dichotomous variables, and the mean difference with 95% CI for continuous variables. Studies were weighed by the inverse of the variance of the outcome. The random-effects model was used for all meta-analyses. Statistical heterogeneity between studies was assumed if *p* < 0.10 for the Cochran’s Chi-square test or *I*^2^ statistic > 50% [15].

### Risk of bias and quality assessment

Two reviewers (AS/PK) scored the quality of the included studies using the methodological items for non-randomized studies (MINORS) [16]. Disagreement between the reviewers was resolved by discussion.

## Results

### Study selection

**Fig. 1:**
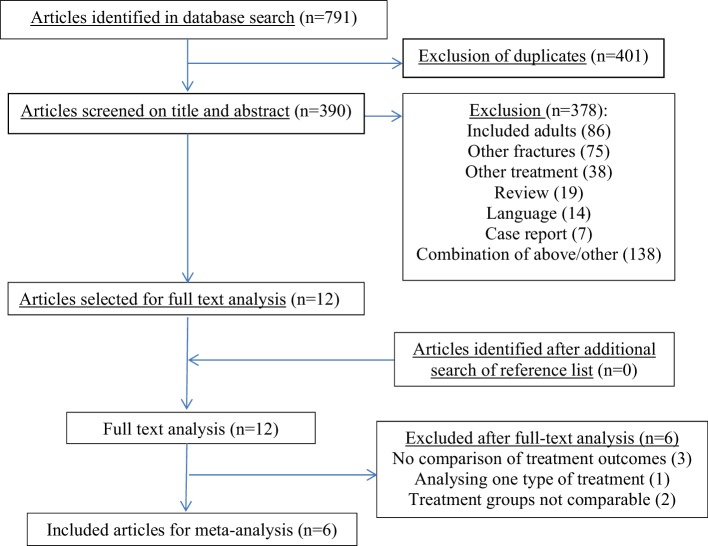
Flowchart of study selection

The search resulted in 791 articles. After exclusion of 401 duplicates, 390 articles remained of which 378 were excluded based on title and abstract. Fourteen of these were excluded based on language, none of which were RCTs or comparative studies. The reference lists of the 12 selected full-text articles were screened for additional studies that were eligible but no other relevant articles were found. After reading the 12 full-text articles, 6 were included in this meta-analysis (Fig. 1).

### Study characteristics

The 6 studies (3 randomised controlled trials, 1 prospective and 2 retrospective cohort studies) described 197 patients who received RCI alone and 185 patients who were treated with additional KWF [9, 13, 17–20]. The studies had a 100% follow-up rate except for the RCTs of McLauchlan et al. (82%) and Colaris et al. (96%). Inclusion and exclusion criteria were similar for al studies. Patients who were treated with cast immobilization alone, were immobilized for a period between 4 and 6 weeks. The duration of cast immobilization depended on the age of the patient and/or visible callus formation on the follow-up radiographs. Surgically treated patients in the studies of Miller et al. and Gibbons et al. had a BEC after removal of the K-wires, for 1–2 weeks [13, 17]. The mean age of the patients included in the studies was 8–13 years, the majority were male. A total of 146 (74.1%) patients treated with RCI and 146 (78.9%) patients with KWF also had a distal ulnar fracture (Table 1).

**Table 1:** Study characteristics

Author	Year of publication	Country	Study design	Evaluated patients Cast alone vs additional K-wire fixation	Follow-up in months, mean (range)	Age in years, mean (± SD or range) Cast alone vs additional K-wire fixation	% male patients Cast alone vs additional K-wire fixation	% patients with concomitant ulnar fracture Cast alone vs additional K-wire fixation	% patients with complete displacement Cast alone vs additional K-wire fixation
Gibbons et al. [13]	1994	United Kingdom	Prospective cohort study	11 vs 12	6	8 (± 6) vs 9 (± 4)	7/11 (63.6%) vs 8/12 (66.7%)	0	8/11 (72.7%) vs 8/12 (75%)
McLauchlan et al. [9]	2002	United Kingdom	RCT	33 vs 35	3	7.6 vs 8.1	20/33 (60.6%) vs 22/35 (62.9%)	28/33 (84.8%) vs 32/35 (91.4%)	100 vs 100%
Miller et al. [17]	2005	USA	RCT	18 vs 16	2.6	12.8 (10–14) vs 12.0 (10–14)	17/18 (94.4%) vs 14/16 (87.5%)	0	100 vs 100%
Ozcan et al. [18]	2010	Turkey	Retrospective cohort study	20 vs 20	20 (6–84)	11.2 (5–15) vs 10.1 (6–14)	–	16/20 (80%) vs 17/20 (85%)	–
Van Egmond et al. [19]	2012	Holland	Retrospective cohort study	48 vs 41	5.8 (1–51)	9.3 (± 3.4) vs 9.2 (± 2.9)	35/48 (72.9%) vs 24/41 (58.5%)	35/48 (72.9%) vs 36/41 (87.8%)	–
Colaris et al. [20]	2013	Holland	RCT	67 vs 61	7.1	8.7 (± 3.2) vs 9.0 (± 3.0)	(42/67) 62.7% vs (41/61) 67.2%	100%	–

### Redisplacement rate

**Fig. 2:**
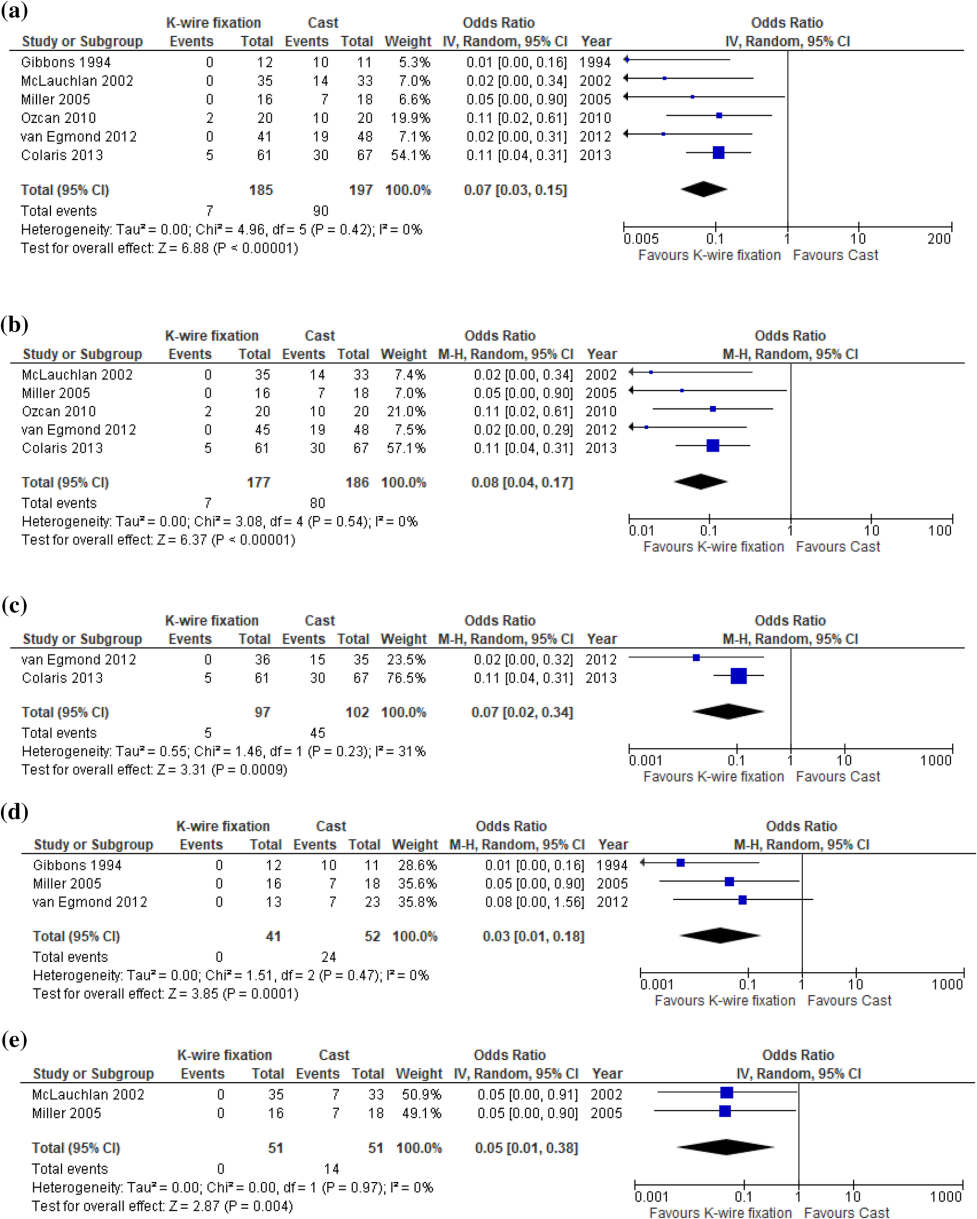
Redisplacement rate; additional K-wire fixation vs cast immobilization alone for **a** all patients with distal radius fractures patients, **b** patients with metaphyseal fractures, **c** patients with both-bone distal fractures, **d** patients with isolated distal radius fractures and **e** patients with completely displaced distal fractures

Definitions for redisplacement differed between the included studies (Table 2). Redisplacement occurred in 3.8% of the patients (7/185) after additional KWF and in 45.7% (90/197) after RCI alone (OR 0.07; 95% CI 0.03–0.15; Fig. 2a). Similar results were found for subgroups of patients with specific types of DRFs (Fig. 2b–e) [9, 17–20]. Five cases of redisplacement occurred in the KWF group in the study by Colaris et al., which were caused by technical errors (n = 2) or redisplacement of the non-fixated ulnar fracture (*n* = 3) [20]. The causes for redisplacement in two patients with additional KWF in the study of Ozcan et al. were not reported [18]. No indication for statistical heterogeneity was found in these meta-analyses.

**Table 2:** Indications for reduction and secondary treatment

Author	Indication for primary reduction	Definition of redisplacement and indication for secondary treatment	Redisplacement^a^	Secondary reduction and cast alone or additional K-wire fixation^a^
Gibbons et al. [13]	Complete displacement Angulation > 10° if > 10 years Angulation > 15° if < 10 years	–	10/11 vs 0/12	10/11 vs 0/12
McLauchlan et al. [9]	Complete displacement	Angulation > 20°, > 50% displacement	14/33 vs 0/35	7/33 vs 0/35^b^
Miller et al. [17]	Complete displacement Angulation > 30°	Angulation > 25°, complete displacement	7/18 vs 0/16	6/18 vs 0/16^b^
Ozcan et al. [18]	> 50% displacement Angulation > 20° if > 10 years Angulation > 30° if < 10 years Bayonet apposition, volar angulation	–	10/20 vs 2/20	0/20 vs 1/20
Van Egmond et al. [19]	> 50% displacement Angulation > 10° if > 10 years Angulation > 15° if < 10 years	–	19/48 vs 0/41	19/48 vs 0/41
Colaris et al. [20]	> 50% displacement Angulation > 10° if > 10 years Angulation > 15° if < 10 years	> 50% displacement Angulation > 10° if > 10 years Angulation > 15° if < 10 years	30/67 vs 5/61	17/67 vs 1/61

^a^Patient numbers: Cast alone vs additional K-wire fixation

^b^One patient wedging of cast

### Secondary reduction

Overall, 61/97 patients (62.9%) with a redisplaced fracture underwent secondary reduction. This concerned 65.6% (59/90) of the patients with redisplacement after RCI alone and 28.6% (2/7) after additional KWF. Between studies, the secondary reduction rate after redisplacement ranged from 0 to 100% (Table 2) [9, 13, 17–20].

### Range of motion

**Fig. 3:**
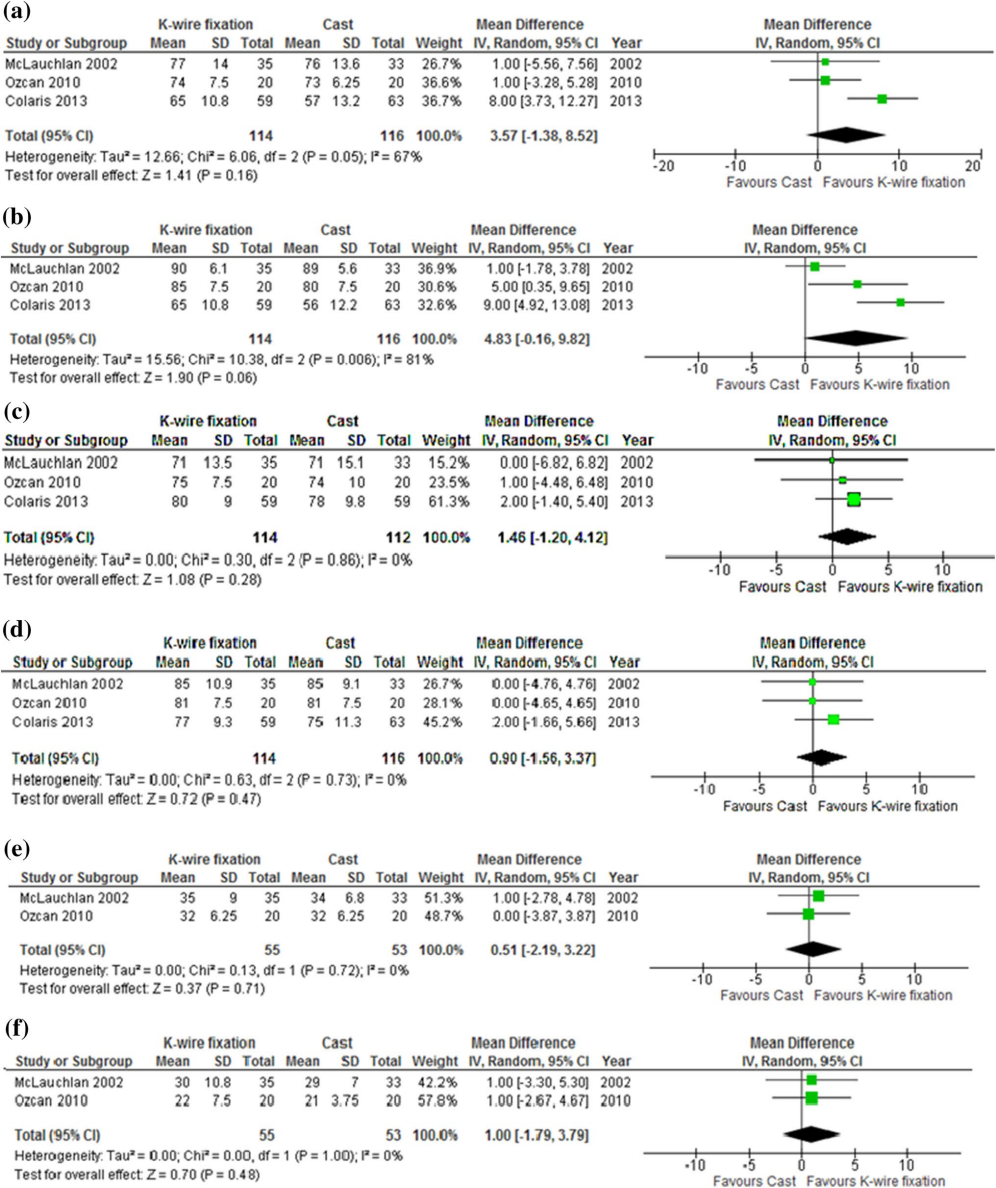
Range of motion in degrees; Mean difference between additional K-wire fixation vs Cast immobilization alone for **a** flexion, **b** extension, **c** pronation, **d** supination, **e** radial deviation, **f** ulnar deviation

Three studies reported the ROM in degrees at final follow-up at 3–20 months [9, 18, 20]. No statistically or clinically significant difference was found for any of the six motions between RCI alone and additional KWF after short and long term follow-up (Fig. 3). Furthermore, Miller et al. reported no limitations in ROM, no alterations in strength or restrictions in activity in both treatment groups after an average follow-up of 2.8 years [17]. Statistical heterogeneity between studies was found for flexion and pronation.

### Complications

Complications other than redisplacement were reported for seven patients (3.6%) after RCI (Table 3). Two patients had malunion of the fracture, one of whom had a corrective osteotomy after 6 months. Twenty-nine patients had minor complications after additional KWF (29/185; 15.7%), most of these were K-wire related. For both treatment groups, no cases of early physeal closure, compartment syndrome, non-union, permanent nerve damage, growth disturbances or complications regarding the anaesthetics were reported [9, 13, 17–20].

**Table 3:** Complications reported after treatment with cast immobilization alone compared to additional K-wire fixation

Complications	Cast alone (*n* = 97/197; 49.2%)	Additional K-wire fixation (*n* = 36/185; 19.4%)
Redisplacement	90/197; 45.7%	7/185; 3.8%
General	(7/197; 3.6%)	(9/185; 4.9%)
Transient neuropraxia	3	2
Refracture	1	4
Malunion^a^	2	–
Prominent scar	1^b^	3
K-wire related	(0/252)	(20/185; 10.8%)
Migrating wire	–	7
Subcutaneous wire^c^	–	7
Infection	–	4
Failed insertion of K-wire	–	1
Tendonitis	–	1

^a^1 loss of position requiring corrective osteotomy after 6 months, the other did not receive further treatment

^b^Scar after pressure sore

^c^Wires were most likely cut too short resulting in subcutaneous wires

### Risk of bias and quality assessment

The risk of bias was assessed using the MINORS criteria (Table 4) [16]. All studies had a clearly stated aim, appropriate follow-up duration and similar study groups. Notable is that only Colaris et al. calculated the needed population size beforehand [20]. Miller et al. included 9 (26%) patients that were not randomized but for whom the treatment choice was based on the preference of the surgeon on call. This resulted in a difference in mean angulation and shortening suggesting more bayonet position in the KWF group before treatment [17]. None of the studies were blinded. However, to minimize bias and inter observer variability, one independent surgeon or physiotherapist assessed the outcomes in three studies [9, 18, 20]. The studies by Ozcan et al., and Van Egmond et al., had the lowest scores for methodological quality. This was mainly due to the retrospective design of these studies [18, 19].

**Table 4:** MINORS scores for the 6 included studies

	Gibbons et al. [13]	McLauchlan et al. [9]	Miller et al. [17]	Ozcan et al. [18]	Van Egmond et al. [19]	Colaris et al. [20]
1. A clearly stated aim	2	2	2	2	2	2
2. Inclusion of consecutive patients	2	2	2	0	2	2
3. Prospective collection of data	2	2	2	1	1	2
4. Endpoints appropriate to the aim of the study	2	2	2	2	2	2
5. Unbiased assessment of the study endpoint	0	0	0	1	0	1
6. Follow-up period appropriate to the aim of the study	2	2	2	2	2	2
7. Loss to follow-up less than 5%	2	1	1	0	0	2
8. Prospective calculation of the study size	0	0	0	0	0	2
9. An adequate control group	2	2	2	2	2	2
10. Contemporary groups	2	2	2	0	2	2
11. Baseline equivalence of groups	2	2	1	2	2	2
12. Adequate statistical analyses	0	2	2	2	2	2
Total	18	19	18	14	17	23

## Discussion

This meta-analysis of six studies aimed to determine whether additional KWF is the preferred treatment for DDRFs in children. The most important finding is that, not surprisingly, in all subgroups the redisplacement rate is considerably lower for fractures treated with KWF. However, complications other than redisplacement, although minor, were more common after additional KWF and mostly K-wire related, such as superficial infection and K-wire migration. The ROM did not differ between RCI alone and additional KWF, including those patients in whom redisplacement occurred after primary treatment and no secondary treatment was performed.

Our results also showed that redisplacement occurs in nearly half of the cases after reduction and cast immobilization alone (90/197; 45.7%). In 65.5% (59/90) of these patients re-reduction was attempted, with rates varying widely between the studies. Indications for secondary interventions after redisplacement were not clearly reported in all studies (Table 2). Colaris et al. stated that all redisplaced fractures should be remanipulated, however, only 56.7% (17/30) of their patients actually received secondary treatment. They suggested that this might be because the surgeon assumed that there was still enough potential for remodelling or did not want to burden the child again with another treatment [20]. Half of the patients with redisplaced fractures in the study of McLauchlan et al. and none of in the study of Ozcan et al. were remanipulated. Neither studies defined the indications for remanipulation, nor did they report why these patients did not receive secondary treatment, but the reasons are expected to be similar to the ones suggested by Colaris et al. [9, 18, 20]. The most common type of secondary treatment was secondary RCI with additional KWF for patients in the study of Van Egmond et al., and secondary RCI alone in the studies of McLauchlan et al. and Miller et al [9, 17, 19]. Two patients had cast wedging to correct redisplacement [9, 17].

Despite treatment differences, the ROM did not differ after 20–34 months [17, 18]. The results of Ozcan et al. showed that even though 50% of the patients with conservative treatment had redisplacement but no secondary treatment, there was no clinically relevant difference in the ROM between the RCI alone and the additional KWF groups [18]. Similar results were found by Colaris et al. Although not quantified, this suggests that considerable angulation or dislocation could be accepted without loss of functionality [9, 20]. These findings are supported by Roth et al. who showed no significant difference in ROM between the different treatment groups (no reduction vs reduction) for paediatric DDRFs after a mean follow-up of 4.0 years [21]. Of course remodelling and thus age of the patients plays an important role in the residual capacity to compensate for the resulting redisplacement at the beginning of the bone-healing process. Additionally, in daily clinical practice the amount of displacement will influence the choice for remanipulation vs a wait-and-see policy. These factors should be considered in each child, although the above described results suggest that even though redisplacement occurs, remanipulation is not always necessary for the final, long-term, function of the wrist.

RCI with additional KWF is a safe treatment option for DDRFs in children and mainly leads to minor complications such as superficial infections and K-wire migration, which may be prevented by not cutting the wires too short and/or using a K-wire pin cover [20]. Although serious complications such as compartment syndrome, permanent nerve damage and early physeal closure after operative treatment potentially leading to growth disturbances did not occur in the included studies, the risk of these complications should still be taken into account [22].

In 5 of the included studies, K-wires were removed under general anaesthesia [9, 13, 17–19]. Colaris et al. however, removed these in an outpatient setting without anaesthetics and did not experience any problems [20]. This procedure is shown not to be very traumatic for the child and a good alternative, with a mean pain VAS-score of 1.4. No difference in VAS-score was found for K-wires of different gauges, duration of stabilisation or anatomical site [23]. Thus, the K-wire removal in an outpatient setting is a suitable option worth considering and discussing with the parents and child.

Finally, financial costs might be considered in the choice of treatment. Additional KWF may be associated with higher costs than RCI alone because of the operative intervention(s). A few studies on this topic were published with conflicting results. A cost analysis by Crawford et al. showed that the costs of additional KWF were almost twice as high compared to RCI alone (8742 vs 4846 dollars) [24]. In an analysis by Miller et al., the costs of additional KWF were also higher, but the difference was much smaller (3150 vs 2750 dollars). However, a more elaborate analysis by Miller et al. showed that, since RCI alone often leads to redisplacement necessitating further intervention, the total costs in this group of patients were actually higher when the complication-related costs were also taken into account [17].

### Study limitations

Only 6 studies could be included due to the limited number of published studies comparing RCI alone and additional KWF for similar patient and fracture related characteristics. Half of the studies were not randomised and the numbers of included patients in most studies were low. However, the inclusion and exclusion criteria for the selected patients in all included studies were similar [9, 13, 17–20].

A second limitation of this meta-analysis is the use of different definitions for redisplacement. Van Egmond et al. for example, defined redisplaced fractures as fractures that required secondary treatment but, as shown in the other included studies, not all redisplaced fractures are remanipulated which can result in an underestimation of the true number of fractures that redisplaced [19]. However, one can question whether the fact that no further intervention was performed on these redisplaced fractures was of any influence on the final function of these children’s arm. In contrast, the other studies had specified definitions with maximum acceptable degrees of angulation and translation, but these were not adjusted for age and limits of remodelling, except in the study of Colaris et al. (Table 2) [9, 13, 17–20]. Because of the lack of these adjustments no conclusions could be drawn separately for young children and older (pre-teen) children. This underlines the ongoing debate about the limits and potential of remodelling in children and the effect of redisplacement. Both should be further defined in relation to outcome, per age group, to further substantiate treatment decisions in children with DDRFs.

## Conclusions

Although this meta-analysis shows that additional KWF leads to a significantly lower redisplacement rate and far less re-interventions than RCI alone for treatment of children with DDRFs, the results also suggest that only RCI is as good as RCI plus KWF regarding functional outcome. This is even so after redisplacement occurs and has been left untreated. These results suggest that larger degrees of angulation and/or displacement could be accepted in children. Future research should preferably identify those that will benefit most from additional KWF, considering the amount of fracture displacement that is acceptable in relation to the age, the subsequent residual remodelling capacity in persistent fracture dislocation and the risk factors for redisplacement in this group of patients.

## Electronic supplementary material

Below is the link to the electronic supplementary material.

Supplementary material 1 (DOCX 15 KB)

## References

[1] Cheng JC, Shen WY (1993). Limb fracture pattern in different pediatric age groups: a study of 3,350 children. J Orthop Trauma.

[2] Worlock P, Stower M (1986). Fracture patterns in Nottingham children. J Pediatr Orthop.

[3] Rennie L (2007). The epidemiology of fractures in children. Injury.

[4] Landin LA (1997). Epidemiology of children’s fractures. J Pediatr Orthop B.

[5] McQuinn AG, Jaarsma RL (2012). Risk factors for redisplacement of pediatric distal forearm and distal radius fractures. J Pediatr Orthop.

[6] Proctor MT, Moore DJ, Paterson JM (1993). Redisplacement after manipulation of distal radial fractures in children. J Bone Jt Surg Br.

[7] Zamzam MM, Khoshhal KI (2005). Displaced fracture of the distal radius in children: factors responsible for redisplacement after closed reduction. J Bone Jt Surg Br.

[8] Asadollahi S, Ooi KS, Hau RC (2015). Distal radial fractures in children: risk factors for redisplacement following closed reduction. J Pediatr Orthop.

[9] McLauchlan GJ (2002). Management of completely displaced metaphyseal fractures of the distal radius in children. A prospective, randomised controlled trial. J Bone Jt Surg Br.

[10] Mostafa MF, El-Adl G, Enan A (2009). Percutaneous Kirschner-wire fixation for displaced distal forearm fractures in children. Acta Orthop Belg.

[11] Choi KY (1995). Percutaneous Kirschner-wire pinning for severely displaced distal radial fractures in children. A report of 157 cases. J Bone Jt Surg Br.

[12] Horii E (1993). Premature closure of the distal radial physis. J Hand Surg Br.

[13] Gibbons CL (1994). The management of isolated distal radius fractures in children. J Pediatr Orthop.

[14] Moher D (2010). Preferred reporting items for systematic reviews and meta-analyses: the PRISMA statement. Int J Surg.

[15] Bown MJ, Sutton AJ (2010). Quality control in systematic reviews and meta-analyses. Eur J Vasc Endovasc Surg.

[16] Slim K (2003). Methodological index for non-randomized studies (minors): development and validation of a new instrument. ANZ J Surg.

[17] Miller BS (2005). Cast immobilization versus percutaneous pin fixation of displaced distal radius fractures in children: a prospective, randomized study. J Pediatr Orthop.

[18] Ozcan M (2010). Percutaneous Kirschner Wire fixation in distal radius metaphyseal fractures in children: does it change the overall outcome?. Hippokratia.

[19] Van Egmond PW, Schipper IB, van Luijt PA (2012). Displaced distal forearm fractures in children with an indication for reduction under general anesthesia should be percutaneously fixated. Eur J Orthop Surg Traumatol.

[20] Colaris JW (2013). Re-displacement of stable distal both-bone forearm fractures in children: a randomised controlled multicentre trial. Injury.

[21] Roth KC (2014). Think twice before re-manipulating distal metaphyseal forearm fractures in children. Arch Orthop Trauma Surg.

[22] Boyden EM, Peterson HA (1991). Partial premature closure of the distal radial physis associated with Kirschner wire fixation. Orthopedics.

[23] Symons S, Persad R, Paterson M (2005). The removal of percutaneous Kirschner wires used in the stabilisation of fractures in children. Acta Orthop Belg.

[24] Crawford SN, Lee LSK, Izuka BH (2012). Closed treatment of overriding distal radial fractures without reduction in children. J Bone Jt Surg Ser A.

