# Supplementary material for: Displaced distal radius fractures in children, cast alone vs additional K-wire fixation: a meta-analysis
Source: Eur J Trauma Emerg Surg. 2018 Oct 1;45(6):1003–11. doi: 10.1007/s00068-018-1011-y (PMC6910898; doi:10.1007/s00068-018-1011-y)
Supplement: Supplementary file 1 — Supplementary material 1 (DOCX 15 KB) [file 68_2018_1011_MOESM1_ESM.docx]

**APPENDIX 1 – Search strategy**

Pubmed: (("displaced distal radius fracture"[tw] OR "displaced distal radius fractures"[tw] OR "displaced distal radial fracture"[tw] OR "displaced distal radial fractures"[tw] OR "displaced distal forearm fractures"[tw] OR (("Radius Fractures"[Mesh] OR "Radius Fractures"[tw] OR "Radius Fracture"[tw] OR "Radial Fractures"[tw] OR "Radial Fracture"[tw] OR "forearm fractures"[tw] OR "forearm fracture"[tw] OR "metaphyseal fractures"[tw] OR "metaphyseal fracture"[tw] OR "wrist fractures"[tw] OR "wrist fracture"[tw]) AND ("dislocated"[tw] OR dislocat*[tw] OR "displaced"[tw] OR displac*[tw] OR "unstable"[tw] OR unstable*[tw] OR "translation"[tw] OR translat*[tw]))) AND (("child"[all fields] NOT child[au]) OR child*[tw] OR children*[tw] OR schoolchild*[tw] OR pediatri*[tw] OR paediatr*[tw] OR boy[tw] OR boys[tw] OR boyhood[tw] OR schoolboy*[tw] OR girl[tw] OR girls[tw] OR girlhood[tw] OR schoolgirl*[tw] OR preteen*[tw] OR puberty[tw] OR preschool*[tw] OR "Adolescent"[Mesh] OR "adolescent"[tw] OR "adolescents"[tw] OR "Adolescence"[tw] OR adolescen*[tw] OR youth[tw] OR youths[tw] OR teen[tw] OR teens[tw] OR teenager*[tw] OR juvenile[tw]) AND ("K-wiring"[tw] OR "K-wire"[tw] OR "K-wires"[tw] OR "Kwiring"[tw] OR "Kwire"[tw] OR "Kwires"[tw] OR "Kirschner-wiring"[tw] OR "kirschner-wire"[tw] OR "Kirschner-wires"[tw] OR kirchner wir*[tw] OR "percutaneous fixation"[tw] OR "percutaneous nail"[tw] OR "percutaneous nails"[tw] OR "percutaneous nailing"[tw] OR "percutaneous wiring"[tw] OR "percutaneous wires"[tw] OR "percutaneous wire"[tw] OR "intramedullary fixation"[tw] OR "intramedullary nail"[tw] OR "intramedullary nails"[tw] OR "intramedullary nailing"[tw] OR "intramedullary wiring"[tw] OR "intramedullary wires"[tw] OR "intramedullary wire"[tw] OR "intramedullary pinning"[tw] OR "intramedullary pin"[tw] OR "intramedullary pins"[tw] OR "osteosynthesis"[tw] OR (transphyseal*[tw] AND fixat*[tw]) OR "Elastic stable intramedullary nailing"[tw] OR "ESIN"[tw] OR "bone wire"[tw] OR "bone wires"[tw] OR "bone wiring"[tw] OR "Bone Wires"[Mesh] OR "Internal Fixators"[mesh:noexp] OR "Bone Nails"[tw] OR Internal Fixat*[tw] OR "Fracture Fixation, Internal"[mesh] OR "Internal Fracture Fixation"[tw] OR "Internal Fracture Fixations"[tw] OR "Fracture Osteosyntheses"[tw] OR "Fracture Osteosynthesis"[tw] OR "Fracture Fixation, Intramedullary"[mesh] OR "Intramedullary Fracture Fixation"[tw] OR "Intramedullary Fracture Fixations"[tw] OR "Intramedullary Nailings"[tw] OR "nail"[tw] OR "nails"[tw] OR "nailing"[tw] OR "wire"[tw] OR "wires"[tw] OR "wiring"[tw]) AND ("Casts, Surgical"[Mesh] OR "cast"[tw] OR "casts"[tw] OR "casting"[tw] OR "plastercast"[tw] OR "plastercasts"[tw] OR "plastercasting"[tw] OR plastercast*[tw] OR "plaster"[tw] OR plaster*[tw] OR reduction[ti] OR "External Fixators"[Mesh] OR External Fixat*[tw] OR "Immobilization"[Mesh] OR "immobilization"[tw] OR "immobilisation"[tw] OR "immobilized"[tw] OR "immobilised"[tw]))

Embase: (("displaced distal radius fracture".mp OR "displaced distal radius fractures".mp OR "displaced distal radial fracture".mp OR "displaced distal radial fractures".mp OR "displaced distal forearm fractures".mp OR (("Radius Fracture"/ OR "Radius Fractures".mp OR "Radius Fracture".mp OR "Radial Fractures".mp OR "Radial Fracture".mp OR "forearm fractures".mp OR "forearm fracture".mp OR "metaphyseal fractures".mp OR "metaphyseal fracture".mp OR "wrist fractures".mp OR "wrist fracture".mp) AND ("fracture dislocation"/ OR "dislocated".mp OR dislocat*.mp OR "displaced".mp OR displac*.mp OR "unstable".mp OR unstable*.mp OR "translation".mp OR translat*.mp))) AND (exp "Child"/ OR child*.mp OR children*.mp OR schoolchild*.mp OR pediatri*.mp OR paediatr*.mp OR boy.mp OR boys.mp OR boyhood.mp OR schoolboy*.mp OR girl.mp OR girls.mp OR girlhood.mp OR schoolgirl*.mp OR preteen*.mp OR puberty.mp OR preschool*.mp OR exp "Adolescent"/ OR "adolescent".mp OR "adolescents".mp OR "Adolescence".mp OR adolescen*.mp OR youth.mp OR youths.mp OR teen.mp OR teens.mp OR teenager*.mp OR juvenile.mp) AND ("Kirschner wire "/ OR "K-wiring".mp OR "K-wire".mp OR "K-wires".mp OR "Kwiring".mp OR "Kwire".mp OR "Kwires".mp OR "Kirschner-wiring".mp OR "kirschner-wire".mp OR "Kirschner-wires".mp OR kirchner wir*.mp OR "percutaneous fixation".mp OR "percutaneous nail".mp OR "percutaneous nails".mp OR "percutaneous nailing".mp OR "percutaneous wiring".mp OR "percutaneous wires".mp OR "percutaneous wire".mp OR "intramedullary fixation".mp OR "intramedullary nail".mp OR "intramedullary nails".mp OR "intramedullary nailing".mp OR "intramedullary wiring".mp OR "intramedullary wires".mp OR "intramedullary wire".mp OR "intramedullary pinning".mp OR "intramedullary pin".mp OR "intramedullary pins".mp OR "osteosynthesis".mp OR (transphyseal*.mp AND fixat*.mp) OR "Elastic stable intramedullary nailing".mp OR "ESIN".mp OR "bone wire".mp OR "bone wires".mp OR "bone wiring".mp OR exp "Bone Wire"/ OR "Internal Fixator"/ OR "Bone Nails".mp OR Internal Fixat*.mp OR "Internal Fracture Fixation".mp OR "Internal Fracture Fixations".mp OR "Fracture Osteosyntheses".mp OR "Fracture Osteosynthesis".mp OR "Intramedullary Fracture Fixation".mp OR "Intramedullary Fracture Fixations".mp OR "Intramedullary Nailings".mp OR "nail".mp OR "nails".mp OR "nailing".mp OR "wire".mp OR "wires".mp OR "wiring".mp OR "compression osteosynthesis"/ OR "intramedullary nailing"/ OR "osteosynthesis"/ OR "wire fixation"/) AND ("orthopedic cast"/ OR "cast".mp OR "casts".mp OR "casting".mp OR "plastercast".mp OR "plastercasts".mp OR "plastercasting".mp OR plastercast*.mp OR "plaster".mp OR plaster*.mp OR reduction.ti OR exp "External Fixator"/ OR External Fixat*.mp OR "fracture external fixation"/ OR "fracture immobilization"/ OR "immobilization".mp OR "immobilisation".mp OR "immobilized".mp OR "immobilised".mp))

Web of Science: TS=(("displaced distal radius fracture" OR "displaced distal radius fractures" OR "displaced distal radial fracture" OR "displaced distal radial fractures" OR "displaced distal forearm fractures" OR (("Radius Fracture" OR "Radius Fractures" OR "Radius Fracture" OR "Radial Fractures" OR "Radial Fracture" OR "forearm fractures" OR "forearm fracture" OR "metaphyseal fractures" OR "metaphyseal fracture" OR "wrist fractures" OR "wrist fracture") AND ("fracture dislocation" OR "dislocated" OR dislocat* OR "displaced" OR displac* OR "unstable" OR unstable* OR "translation" OR translat*))) AND ("Child" OR child* OR children* OR schoolchild* OR pediatri* OR paediatr* OR boy OR boys OR boyhood OR schoolboy* OR girl OR girls OR girlhood OR schoolgirl* OR preteen* OR puberty OR preschool* OR "Adolescent" OR "adolescent" OR "adolescents" OR "Adolescence" OR adolescen* OR youth OR youths OR teen OR teens OR teenager* OR juvenile) AND ("Kirschner wire " OR "K-wiring" OR "K-wire" OR "K-wires" OR "Kwiring" OR "Kwire" OR "Kwires" OR "Kirschner-wiring" OR "kirschner-wire" OR "Kirschner-wires" OR kirchner wir* OR "percutaneous fixation" OR "percutaneous nail" OR "percutaneous nails" OR "percutaneous nailing" OR "percutaneous wiring" OR "percutaneous wires" OR "percutaneous wire" OR "intramedullary fixation" OR "intramedullary nail" OR "intramedullary nails" OR "intramedullary nailing" OR "intramedullary wiring" OR "intramedullary wires" OR "intramedullary wire" OR "intramedullary pinning" OR "intramedullary pin" OR "intramedullary pins" OR "osteosynthesis" OR (transphyseal* AND fixat*) OR "Elastic stable intramedullary nailing" OR "ESIN" OR "bone wire" OR "bone wires" OR "bone wiring" OR "Bone Wire" OR "Internal Fixator" OR "Bone Nails" OR Internal Fixat* OR "Internal Fracture Fixation" OR "Internal Fracture Fixations" OR "Fracture Osteosyntheses" OR "Fracture Osteosynthesis" OR "Intramedullary Fracture Fixation" OR "Intramedullary Fracture Fixations" OR "Intramedullary Nailings" OR "nail" OR "nails" OR "nailing" OR "wire" OR "wires" OR "wiring" OR "compression osteosynthesis" OR "intramedullary nailing" OR "osteosynthesis" OR "wire fixation") AND ("orthopedic cast" OR "cast" OR "casts" OR "casting" OR "plastercast" OR "plastercasts" OR "plastercasting" OR plastercast* OR "plaster" OR plaster* OR reduction.ti OR "External Fixator" OR External Fixat* OR "fracture external fixation" OR "fracture immobilization" OR "immobilization" OR "immobilisation" OR "immobilized" OR "immobilised"))

Cochrane: (("displaced distal radius fracture" OR "displaced distal radius fractures" OR "displaced distal radial fracture" OR "displaced distal radial fractures" OR "displaced distal forearm fractures" OR (("Radius Fracture" OR "Radius Fractures" OR "Radius Fracture" OR "Radial Fractures" OR "Radial Fracture" OR "forearm fractures" OR "forearm fracture" OR "metaphyseal fractures" OR "metaphyseal fracture" OR "wrist fractures" OR "wrist fracture") AND ("fracture dislocation" OR "dislocated" OR dislocat* OR "displaced" OR displac* OR "unstable" OR unstable* OR "translation" OR translat*))) AND ("Child" OR child* OR children* OR schoolchild* OR pediatri* OR paediatr* OR boy OR boys OR boyhood OR schoolboy* OR girl OR girls OR girlhood OR schoolgirl* OR preteen* OR puberty OR preschool* OR "Adolescent" OR "adolescent" OR "adolescents" OR "Adolescence" OR adolescen* OR youth OR youths OR teen OR teens OR teenager* OR juvenile) AND ("Kirschner wire " OR "K-wiring" OR "K-wire" OR "K-wires" OR "Kwiring" OR "Kwire" OR "Kwires" OR "Kirschner-wiring" OR "kirschner-wire" OR "Kirschner-wires" OR kirchner wir* OR "percutaneous fixation" OR "percutaneous nail" OR "percutaneous nails" OR "percutaneous nailing" OR "percutaneous wiring" OR "percutaneous wires" OR "percutaneous wire" OR "intramedullary fixation" OR "intramedullary nail" OR "intramedullary nails" OR "intramedullary nailing" OR "intramedullary wiring" OR "intramedullary wires" OR "intramedullary wire" OR "intramedullary pinning" OR "intramedullary pin" OR "intramedullary pins" OR "osteosynthesis" OR (transphyseal* AND fixat*) OR "Elastic stable intramedullary nailing" OR "ESIN" OR "bone wire" OR "bone wires" OR "bone wiring" OR "Bone Wire" OR "Internal Fixator" OR "Bone Nails" OR Internal Fixat* OR "Internal Fracture Fixation" OR "Internal Fracture Fixations" OR "Fracture Osteosyntheses" OR "Fracture Osteosynthesis" OR "Intramedullary Fracture Fixation" OR "Intramedullary Fracture Fixations" OR "Intramedullary Nailings" OR "nail" OR "nails" OR "nailing" OR "wire" OR "wires" OR "wiring" OR "compression osteosynthesis" OR "intramedullary nailing" OR "osteosynthesis" OR "wire fixation") AND ("orthopedic cast" OR "cast" OR "casts" OR "casting" OR "plastercast" OR "plastercasts" OR "plastercasting" OR plastercast* OR "plaster" OR plaster* OR reduction.ti OR "External Fixator" OR External Fixat* OR "fracture external fixation" OR "fracture immobilization" OR "immobilization" OR "immobilisation" OR "immobilized" OR "immobilised"))

CENTRAL: (("displaced distal radius fracture" OR "displaced distal radius fractures" OR "displaced distal radial fracture" OR "displaced distal radial fractures" OR "displaced distal forearm fractures" OR (("Radius Fracture" OR "Radius Fractures" OR "Radius Fracture" OR "Radial Fractures" OR "Radial Fracture" OR "forearm fractures" OR "forearm fracture" OR "metaphyseal fractures" OR "metaphyseal fracture" OR "wrist fractures" OR "wrist fracture") AND ("fracture dislocation" OR "dislocated" OR dislocat* OR "displaced" OR displac* OR "unstable" OR unstable* OR "translation" OR translat*))) AND ("Child" OR child* OR children* OR schoolchild* OR pediatri* OR paediatr* OR boy OR boys OR boyhood OR schoolboy* OR girl OR girls OR girlhood OR schoolgirl* OR preteen* OR puberty OR preschool* OR "Adolescent" OR "adolescent" OR "adolescents" OR "Adolescence" OR adolescen* OR youth OR youths OR teen OR teens OR teenager* OR juvenile) AND ("Kirschner wire " OR "K-wiring" OR "K-wire" OR "K-wires" OR "Kwiring" OR "Kwire" OR "Kwires" OR "Kirschner-wiring" OR "kirschner-wire" OR "Kirschner-wires" OR kirchner wir* OR "percutaneous fixation" OR "percutaneous nail" OR "percutaneous nails" OR "percutaneous nailing" OR "percutaneous wiring" OR "percutaneous wires" OR "percutaneous wire" OR "intramedullary fixation" OR "intramedullary nail" OR "intramedullary nails" OR "intramedullary nailing" OR "intramedullary wiring" OR "intramedullary wires" OR "intramedullary wire" OR "intramedullary pinning" OR "intramedullary pin" OR "intramedullary pins" OR "osteosynthesis" OR (transphyseal* AND fixat*) OR "Elastic stable intramedullary nailing" OR "ESIN" OR "bone wire" OR "bone wires" OR "bone wiring" OR "Bone Wire" OR "Internal Fixator" OR "Bone Nails" OR Internal Fixat* OR "Internal Fracture Fixation" OR "Internal Fracture Fixations" OR "Fracture Osteosyntheses" OR "Fracture Osteosynthesis" OR "Intramedullary Fracture Fixation" OR "Intramedullary Fracture Fixations" OR "Intramedullary Nailings" OR "nail" OR "nails" OR "nailing" OR "wire" OR "wires" OR "wiring" OR "compression osteosynthesis" OR "intramedullary nailing" OR "osteosynthesis" OR "wire fixation") AND ("orthopedic cast" OR "cast" OR "casts" OR "casting" OR "plastercast" OR "plastercasts" OR "plastercasting" OR plastercast* OR "plaster" OR plaster* OR reduction.ti OR "External Fixator" OR External Fixat* OR "fracture external fixation" OR "fracture immobilization" OR "immobilization" OR "immobilisation" OR "immobilized" OR "immobilised"))

CINAHL: (("displaced distal radius fracture" OR "displaced distal radius fractures" OR "displaced distal radial fracture" OR "displaced distal radial fractures" OR "displaced distal forearm fractures" OR (("Radius Fracture" OR "Radius Fractures" OR "Radius Fracture" OR "Radial Fractures" OR "Radial Fracture" OR "forearm fractures" OR "forearm fracture" OR "metaphyseal fractures" OR "metaphyseal fracture" OR "wrist fractures" OR "wrist fracture") AND ("fracture dislocation" OR "dislocated" OR dislocat* OR "displaced" OR displac* OR "unstable" OR unstable* OR "translation" OR translat*))) AND ("Child" OR child* OR children* OR schoolchild* OR pediatri* OR paediatr* OR boy OR boys OR boyhood OR schoolboy* OR girl OR girls OR girlhood OR schoolgirl* OR preteen* OR puberty OR preschool* OR "Adolescent" OR "adolescent" OR "adolescents" OR "Adolescence" OR adolescen* OR youth OR youths OR teen OR teens OR teenager* OR juvenile) AND ("Kirschner wire " OR "K-wiring" OR "K-wire" OR "K-wires" OR "Kwiring" OR "Kwire" OR "Kwires" OR "Kirschner-wiring" OR "kirschner-wire" OR "Kirschner-wires" OR kirchner wir* OR "percutaneous fixation" OR "percutaneous nail" OR "percutaneous nails" OR "percutaneous nailing" OR "percutaneous wiring" OR "percutaneous wires" OR "percutaneous wire" OR "intramedullary fixation" OR "intramedullary nail" OR "intramedullary nails" OR "intramedullary nailing" OR "intramedullary wiring" OR "intramedullary wires" OR "intramedullary wire" OR "intramedullary pinning" OR "intramedullary pin" OR "intramedullary pins" OR "osteosynthesis" OR (transphyseal* AND fixat*) OR "Elastic stable intramedullary nailing" OR "ESIN" OR "bone wire" OR "bone wires" OR "bone wiring" OR "Bone Wire" OR "Internal Fixator" OR "Bone Nails" OR Internal Fixat* OR "Internal Fracture Fixation" OR "Internal Fracture Fixations" OR "Fracture Osteosyntheses" OR "Fracture Osteosynthesis" OR "Intramedullary Fracture Fixation" OR "Intramedullary Fracture Fixations" OR "Intramedullary Nailings" OR "nail" OR "nails" OR "nailing" OR "wire" OR "wires" OR "wiring" OR "compression osteosynthesis" OR "intramedullary nailing" OR "osteosynthesis" OR "wire fixation") AND ("orthopedic cast" OR "cast" OR "casts" OR "casting" OR "plastercast" OR "plastercasts" OR "plastercasting" OR plastercast* OR "plaster" OR plaster* OR reduction.ti OR "External Fixator" OR External Fixat* OR "fracture external fixation" OR "fracture immobilization" OR "immobilization" OR "immobilisation" OR "immobilized" OR "immobilised"))

Academic Search Premier: (("displaced distal radius fracture" OR "displaced distal radius fractures" OR "displaced distal radial fracture" OR "displaced distal radial fractures" OR "displaced distal forearm fractures" OR (("Radius Fracture" OR "Radius Fractures" OR "Radius Fracture" OR "Radial Fractures" OR "Radial Fracture" OR "forearm fractures" OR "forearm fracture" OR "metaphyseal fractures" OR "metaphyseal fracture" OR "wrist fractures" OR "wrist fracture") AND ("fracture dislocation" OR "dislocated" OR dislocat* OR "displaced" OR displac* OR "unstable" OR unstable* OR "translation" OR translat*))) AND ("Child" OR child* OR children* OR schoolchild* OR pediatri* OR paediatr* OR boy OR boys OR boyhood OR schoolboy* OR girl OR girls OR girlhood OR schoolgirl* OR preteen* OR puberty OR preschool* OR "Adolescent" OR "adolescent" OR "adolescents" OR "Adolescence" OR adolescen* OR youth OR youths OR teen OR teens OR teenager* OR juvenile) AND ("Kirschner wire " OR "K-wiring" OR "K-wire" OR "K-wires" OR "Kwiring" OR "Kwire" OR "Kwires" OR "Kirschner-wiring" OR "kirschner-wire" OR "Kirschner-wires" OR kirchner wir* OR "percutaneous fixation" OR "percutaneous nail" OR "percutaneous nails" OR "percutaneous nailing" OR "percutaneous wiring" OR "percutaneous wires" OR "percutaneous wire" OR "intramedullary fixation" OR "intramedullary nail" OR "intramedullary nails" OR "intramedullary nailing" OR "intramedullary wiring" OR "intramedullary wires" OR "intramedullary wire" OR "intramedullary pinning" OR "intramedullary pin" OR "intramedullary pins" OR "osteosynthesis" OR (transphyseal* AND fixat*) OR "Elastic stable intramedullary nailing" OR "ESIN" OR "bone wire" OR "bone wires" OR "bone wiring" OR "Bone Wire" OR "Internal Fixator" OR "Bone Nails" OR Internal Fixat* OR "Internal Fracture Fixation" OR "Internal Fracture Fixations" OR "Fracture Osteosyntheses" OR "Fracture Osteosynthesis" OR "Intramedullary Fracture Fixation" OR "Intramedullary Fracture Fixations" OR "Intramedullary Nailings" OR "nail" OR "nails" OR "nailing" OR "wire" OR "wires" OR "wiring" OR "compression osteosynthesis" OR "intramedullary nailing" OR "osteosynthesis" OR "wire fixation") AND ("orthopedic cast" OR "cast" OR "casts" OR "casting" OR "plastercast" OR "plastercasts" OR "plastercasting" OR plastercast* OR "plaster" OR plaster* OR reduction.ti OR "External Fixator" OR External Fixat* OR "fracture external fixation" OR "fracture immobilization" OR "immobilization" OR "immobilisation" OR "immobilized" OR "immobilised"))

ScienceDirect: TITLE-ABSTR-KEY(("displaced distal radius fracture" OR "displaced distal radius fractures" OR "displaced distal radial fracture" OR "displaced distal radial fractures" OR "displaced distal forearm fractures" OR (("Radius Fracture" OR "Radius Fractures" OR "Radius Fracture" OR "Radial Fractures" OR "Radial Fracture" OR "forearm fractures" OR "forearm fracture" OR "metaphyseal fractures" OR "metaphyseal fracture" OR "wrist fractures" OR "wrist fracture") AND ("fracture dislocation" OR "dislocated" OR dislocat* OR "displaced" OR displac* OR "unstable" OR unstable* OR "translation" OR translat*))) AND ("Child" OR child* OR children* OR schoolchild* OR pediatri* OR paediatr* OR boy OR boys OR boyhood OR schoolboy* OR girl OR girls OR girlhood OR schoolgirl* OR preteen* OR puberty OR preschool* OR "Adolescent" OR "adolescent" OR "adolescents" OR "Adolescence" OR adolescen* OR youth OR youths OR teen OR teens OR teenager* OR juvenile) AND ("Kirschner wire " OR "K-wiring" OR "K-wire" OR "K-wires" OR "Kwiring" OR "Kwire" OR "Kwires" OR "Kirschner-wiring" OR "kirschner-wire" OR "Kirschner-wires" OR kirchner wir* OR "percutaneous fixation" OR "percutaneous nail" OR "percutaneous nails" OR "percutaneous nailing" OR "percutaneous wiring" OR "percutaneous wires" OR "percutaneous wire" OR "intramedullary fixation" OR "intramedullary nail" OR "intramedullary nails" OR "intramedullary nailing" OR "intramedullary wiring" OR "intramedullary wires" OR "intramedullary wire" OR "intramedullary pinning" OR "intramedullary pin" OR "intramedullary pins" OR "osteosynthesis" OR (transphyseal* AND fixat*) OR "Elastic stable intramedullary nailing" OR "ESIN" OR "bone wire" OR "bone wires" OR "bone wiring" OR "Bone Wire" OR "Internal Fixator" OR "Bone Nails" OR Internal Fixat* OR "Internal Fracture Fixation" OR "Internal Fracture Fixations" OR "Fracture Osteosyntheses" OR "Fracture Osteosynthesis" OR "Intramedullary Fracture Fixation" OR "Intramedullary Fracture Fixations" OR "Intramedullary Nailings" OR "nail" OR "nails" OR "nailing" OR "wire" OR "wires" OR "wiring" OR "compression osteosynthesis" OR "intramedullary nailing" OR "osteosynthesis" OR "wire fixation") AND ("orthopedic cast" OR "cast" OR "casts" OR "casting" OR "plastercast" OR "plastercasts" OR "plastercasting" OR plastercast* OR "plaster" OR plaster* OR reduction.ti OR "External Fixator" OR External Fixat* OR "fracture external fixation" OR "fracture immobilization" OR "immobilization" OR "immobilisation" OR "immobilized" OR "immobilised"))
